# Neural Cross-Frequency Coupling Functions

**DOI:** 10.3389/fnsys.2017.00033

**Published:** 2017-06-15

**Authors:** Tomislav Stankovski, Valentina Ticcinelli, Peter V. E. McClintock, Aneta Stefanovska

**Affiliations:** ^1^Nonlinear and Biomedical Physics Group, Department of Physics, Lancaster UniversityLancaster, United Kingdom; ^2^Faculty of Medicine, Ss Cyril and Methodius UniversitySkopje, Macedonia

**Keywords:** coupling function, cross-frequency coupling, dynamical Bayesian inference, effective connectivity, EEG, neural oscillations, resting brain, eyes-open

## Abstract

Although neural interactions are usually characterized only by their coupling strength and directionality, there is often a need to go beyond this by establishing the functional mechanisms of the interaction. We introduce the use of dynamical Bayesian inference for estimation of the coupling functions of neural oscillations in the presence of noise. By grouping the partial functional contributions, the coupling is decomposed into its functional components and its most important characteristics—strength and form—are quantified. The method is applied to characterize the δ-to-α phase-to-phase neural coupling functions from electroencephalographic (EEG) data of the human resting state, and the differences that arise when the eyes are either open (EO) or closed (EC) are evaluated. The δ-to-α phase-to-phase coupling functions were reconstructed, quantified, compared, and followed as they evolved in time. Using phase-shuffled surrogates to test for significance, we show how the strength of the direct coupling, and the similarity and variability of the coupling functions, characterize the EO and EC states for different regions of the brain. We confirm an earlier observation that the direct coupling is stronger during EC, and we show for the first time that the coupling function is significantly less variable. Given the current understanding of the effects of e.g., aging and dementia on δ-waves, as well as the effect of cognitive and emotional tasks on α-waves, one may expect that new insights into the neural mechanisms underlying certain diseases will be obtained from studies of coupling functions. In principle, any pair of coupled oscillations could be studied in the same way as those shown here.

## 1. Introduction

The complexity of the human brain makes its function exceptionally challenging to analyse and understand. Its electrophysiological activity emanates from the dynamics of large-scale cell ensembles (Traub et al., 1996; Klausberger et al., 2003; Breakspear et al., 2010) which oscillate synchronously within characteristic frequency intervals. The ensembles communicate with each other to integrate their local information flows into a common brain network. Arguably, one of the most promising ways of describing communication of that kind is through cross-frequency coupling, and there has been a large number of such studies in recent years to elucidate the functional activity of the brain underlying e.g., cognition, attention, learning and working memory (Jensen and Colgin, 2007; Musizza et al., 2007; Stam et al., 2009; Axmacher et al., 2010; Belluscio et al., 2012; Jirsa and Müller, 2013; Purdon et al., 2013; van Wijk et al., 2013; Blain-Moraes et al., 2015; Sotero, 2016).

The different types of cross-frequency coupling (Jensen and Colgin, 2007; Canolty and Knight, 2010; Voytek et al., 2010; Jirsa and Müller, 2013) depend on the dynamical properties of the oscillating systems that are coupled, e.g., phase, amplitude/power and frequency. The most studied to date in brain dynamics have been the phase-to-phase (Varela et al., 2001) and phase-to-power (Canolty et al., 2006) cross-frequency couplings. The θ-γ coupling has attracted considerable attention and its neurophysiological correlates, especially those related to the working memory (Axmacher et al., 2010; Belluscio et al., 2012), have been largely understood; there are relatively fewer studies of the coupling between δ and α waves (Jirsa and Müller, 2013). These types of investigation are usually based on the statistics of the cross-frequency relationship e.g., in terms of correlation or phase-locking, or on a quantification of the coupling amplitude. Not much has yet been done, however, to assess systematically, *in vivo*, the coupling functions that describe the *functional forms* of individual cross-frequency interactions between neural oscillations.

Coupling functions describe in great detail the physical rule specifying how the interactions occur and manifest themselves. The coupling function as a whole can be described in terms of its strength and form. It is the functional form that has provided the new dimension and perspective on which we focus below. It probes directly the functional *mechanisms* of the interactions. In this way the coupling function can determine the possibility of qualitative transitions between states of the composite system e.g., routes into and out of synchronization, thus playing an active role in the possible self-organization of the systems. Decomposition of a coupling function can also facilitate a description of the functional contributions from each separate subsystem within the coupling relationship.

Recent progress directed toward the extraction and reconstruction of the coupling functions between interacting oscillatory processes has led to a diversity of applications. These include chemical interactions (Kiss et al., 2005; Miyazaki and Kinoshita, 2006; Tokuda et al., 2007), cardiorespiratory interactions (Stankovski et al., 2012; Iatsenko et al., 2013; Kralemann et al., 2013), mechanical interactions (Kralemann et al., 2008), social sciences (Ranganathan et al., 2014) and secure communications (Stankovski et al., 2014b). The study of coupling functions is a very active and expanding field of research (Stankovski et al., 2017). In this paper we evaluate coupling functions between brain waves. We focus on δ-to-α phase-to-phase interactions during eyes opened and closed and illustrate the underlying methodology. Moreover, we clearly show the difference in form of the coupling function between these two states, thereby paving the way to further applications and advancing the understanding of brain function.

## 2. Materials and methods

### 2.1. Wavelet spectral analysis

We computed the wavelet transform (WT) (Kaiser, 1994; Bračič and Stefanovska, 1998; Stefanovska et al., 1999) in order to evaluate the power content within the 0.8–40 Hz range, converting the signals *s*(*t*) to the time-frequency domain:

(1) $$\begin{array}{r} WT\left(\omega ,t\right)=\int_{0}^{\infty }\psi \left(\omega \left(u-t\right)\right)s\left(u\right)\omega \text{d}u, \end{array}$$

where ω denotes angular frequency, *t* is time, and ψ(*u*) = 1/(2π) $\left(e^{\left(i2\pi f_{0}u\right)}-e^{{\left(2\pi f_{0}\right)}^{2}/2}\right)e^{-u^{2}/2}$ (with ∫ ψ(*t*)*dt* = 0) with central frequency *f*_0_ = 1. The power within each frequency interval was assessed by averaging the spectra over the corresponding frequency ranges.

### 2.2. Model of phase dynamics

Amplitude dynamics in living systems is often multidimensional, which can create complications in analysis. In contrast, the phase dynamics of a periodic process in such systems is describable in terms of a single-dimensional observable, which is usually much easier to detect and extract from data. It is well known that brain activity carries the signatures of several distinct neural oscillations that manifest themselves within characteristic frequency intervals (Buzsáki and Draguhn, 2004). The signals extracted from these intervals are periodic, enabling the underlying oscillatory processes and their interactions to be studied effectively through phase dynamics (Kuramoto, 1984), and leading to extraction of phase-to-phase cross-frequency couplings (Jensen and Colgin, 2007; Jirsa and Müller, 2013). The cross-frequency phase couplings coexist in a multivariate and multidimensional space, so we will consider a network model of *N* coupled phase oscillators, each described by

(2) $$\begin{array}{r} \begin{array}{c} {\dot{\phi }}_{i}\left(t\right)=\omega_{i}\left(t\right)+q_{i}\left(\phi_{i},\phi_{j},\phi_{k},\ldots ,\phi_{N},t\right)+\xi_{i}\left(t\right)\\ =\omega_{i}\left(t\right)+\underset{n}{\sum }q_{i}^{\left(1\right)}\left(\phi_{n},t\right)+\underset{nm}{\sum }q_{i}^{\left(2\right)}\left(\phi_{n},\phi_{m},t\right)\\ +\underset{nml}{\sum }q_{i}^{\left(3\right)}\left(\phi_{n},\phi_{m},\phi_{l},t\right)+\ldots +\xi_{i}\left(t\right), \end{array} \end{array}$$

for all *l, m, n*, …, where ${\dot{\phi }}_{i}\left(t\right)$ is the time derivative of the phase (i.e., the instantaneous frequency), ω_*i*_(*t*) is the natural frequency and the external stochastic dynamics ξ_*i*_(*t*) is treated as Gaussian white noise 〈ξ_*i*_(*t*)ξ_*j*_(τ)ξ_*k*_(τ)…〉 = δ(*t* − τ)*D*_*ijk*…_, where *D* is the matrix of noise diffusion and *D*_*i, j, k*.._ gives the noise strength for the particular *i, j, k*… element. Although we will discuss the inference of neural coupling functions from phase dynamics, the method that we will describe is in principle also applicable to their inference from amplitude dynamics (Stankovski et al., 2014b).

The coupling functions $q_{i}^{\left(\kappa \right)}$ describe the dynamics in terms of the phases of κ interacting oscillators. As can be seen from Equation (2), the coupling functions *q*_*i*_ act in such a way as to modify the natural frequency ω_*i*_(*t*): in physical terms, a positive coupling coefficient will accelerate the oscillation in question (by increasing its instantaneous frequency ${\dot{\phi }}_{i}\left(t\right)$), whilst a negative coupling coefficient will decelerate it (by decreasing ${\dot{\phi }}_{i}\left(t\right)$). Thus a coupling function is able to describe in detail, within a single cycle, *how one oscillator is accelerated or decelerated as a result of the influence from the other oscillators*. This carries important implications for the interpretation of the mechanisms underlying the coupling functions, as will be discussed below. Each function $q_{i}^{\left(\kappa \right)}$ is periodic, for κ ≥ 2 on the κ-dimensional torus, and can be decomposed into a sum of κ-dimensional Fourier series of trigonometric functions. In practice it is assumed that the dynamics can be well-described by a finite number *K* of Fourier terms (Kralemann et al., 2011; Duggento et al., 2012): ${\dot{\phi }}_{i}=\overset{K}{\underset{k=-K}{\sum }}c_{k}^{\left(i\right)}\Phi_{i,k}\left(\phi_{1},\phi_{2},\ldots ,\phi_{N}\right)+\xi_{i}=\overset{K}{\underset{k=-K}{\sum }}c_{k}^{\left(i\right)}\exp\left[i\left(k_{1}\phi_{1}+k_{2}\phi_{2}+\ldots +k_{N}\phi_{N}\right)\right]+\xi_{i}$, where *i* = 1, …, *N*, Φ_*i*, 0_ = 1 so that $c_{0}^{\left(l\right)}=\omega_{l}$, and the rest of Φ_*i, k*_, scaled by $c_{k}^{\left(i\right)}$, are the *k* most important Fourier components. Such Fourier series Φ_*i, k*_(ϕ_1_, ϕ_2_, …, ϕ_*N*_) act as base functions for the dynamical inference method.

### 2.3. Dynamical inference

Our aim is to reconstruct a dynamical model describing the interactions through the analysis of data, so that the model can then be used for extraction of the coupling functions. Our approach is based on the method of *dynamical inference*, often referred to as *dynamical modeling* or *dynamical filtering* (Kalman, 1960; Sanjeev Arulampalam et al., 2002; Friston et al., 2003; Voss et al., 2004; von Toussaint, 2011).

Note that inference of cross-frequency couplings from the statistics of the coupled signals, e.g., through correlation, (bi-)coherence and Granger causality measures (Geweke, 1982; Baccala and Sameshima, 2001; Kamiński et al., 2001), yields the *functional connectivity* but it provides no information about the mechanisms of causality. These latter methods are designed to infer statistical effects rather than dynamical mechanisms (Barrett and Barnett, 2013). In what follows, however, with the aid of dynamical inference we discuss how the mechanisms of the associated causality can be inferred from data, thus yielding an *effective connectivity* (Friston, 2011).

In particular, coupling functions represent one type of dynamical mechanism and their inference yields the effective connectivity. More specifically, the form of the coupling function defines the functional law under which some input of the interactions (i.e., the mutual influence between the oscillations) is translated into an appropriate output. This is related, not only to the quantitative parameters of the net coupling strength i.e., net information flow, but also to how this information is functionally structured to give an effective mechanism. For example, as we will see below, the interactions can be such that the *form of the coupling function varies* in time (see e.g., Section 3.3.2 and Stankovski et al., 2012). This dynamical change can cause a qualitative transition (like synchronization), irrespectively of the value and the variations of the net coupling strength. This is an example of a case where functional connectivity methods (e.g., Granger causality) will detect only the net coupling strength and not the possible reason for a qualitative transition, unlike coupling functions analysis which can do so (see below).

A number of different techniques are available for estimating a model from data, based on different procedures and theories, and resulting in slightly different properties and characteristics. They include e.g., least-squares and kernel smoothing fits (Rosenblum and Pikovsky, 2001; Kralemann et al., 2013), dynamical Bayesian inference (Smelyanskiy et al., 2005; Stankovski et al., 2012), maximum likelihood (multiple-shooting) methods (Voss et al., 2004; Tokuda et al., 2007), and dynamic causal modeling (Friston et al., 2003).

In what follows we use the dynamical Bayesian inference technique (Smelyanskiy et al., 2005; Stankovski et al., 2012). Briefly, the method applies Bayesian probability theory to the multidimensional time-series to infer the dynamical model in terms of stochastic differential equations. Assuming a normal multivariate distribution for the prior of the scale parameters, by the use of the model base functions, the method constructs a log-likelihood function which also ensures that the posterior probability is normally distributed. Evaluation of the current distribution relies on the evaluation of the previous block of data in the sequence, i.e., informative priors are used and the current prior depends on the previous posterior. For the first time window, in the absence of an earlier block, we set the initial prior to a flat (zero) distribution; which might effect the precision with which the initial coupling function is inferred for that window. To account for the time-variability of the interacting dynamics, the covariance matrix of the next prior is the convolution of the current posterior with the current diffusion matrix which describes how much the parameters can change. Further details of the method can be found in the Supplementary Material, in Smelyanskiy et al. (2005), Stankovski et al. (2012), Duggento et al. (2012), and Stankovski et al. (2014a) and in the references therein.

### 2.4. Coupling quantifications and decomposition

**Figure 1:**
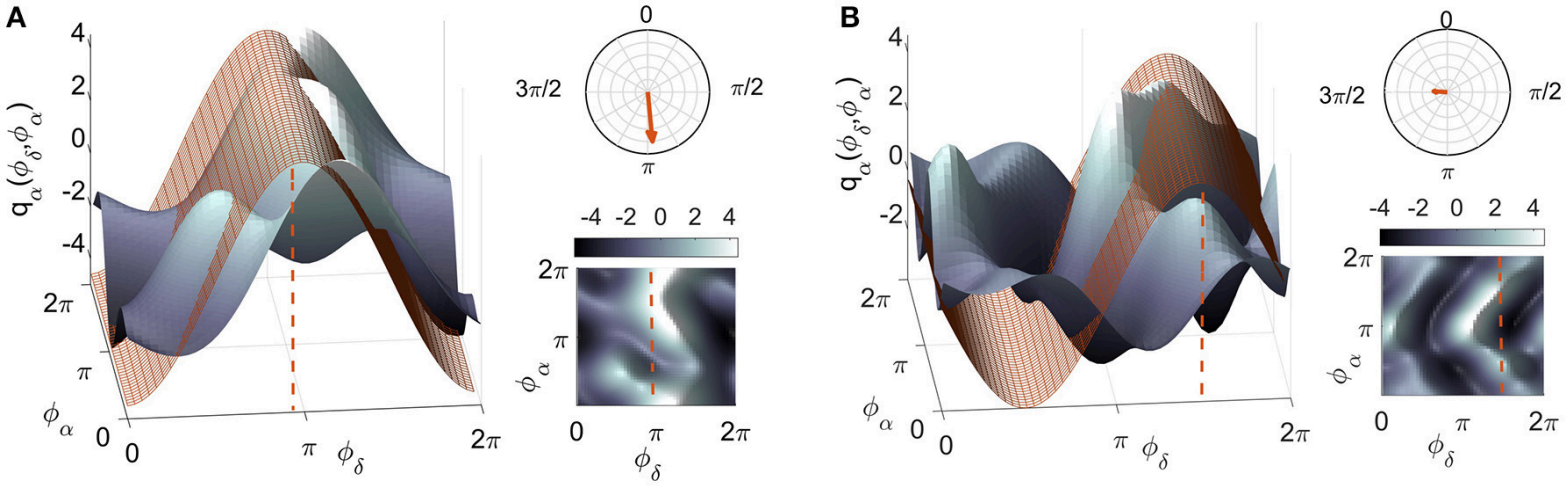
The meaning of the polar similarity index. Two examples of coupling functions, plotted in blue, are compared with numerically-generated sinusoidal functions, plotted in red. The latter have been selected for being as similar as possible to the coupling functions: the only degree of freedom in the selection was the shift in phase (marked by the red dashed lines). The arrows in the polar planes in the top right corners have moduli equal to the similarity indices, and point to the corresponding phase values for: **(A)** a coupling function with high similarity (ρ = 0.82) and **(B)** one with a low value (ρ = 0.23). A complementary 2D color-contour plot of the coupling function is given in the bottom right-hand corner of each panel.

Using the inferred parameters we can calculate the coupling quantities and characteristics. The coupling functions *q*_*i*_(ϕ_*i*_, ϕ_*j*_, ϕ_*k*_, …, ϕ_*N*_) acting on the oscillator from each of the *i* phases are evaluated on a 2π × 2π × … × 2π grid by selecting the relevant base functions, i.e., Fourier components scaled by the corresponding inferred coupling parameters. The coupling strength is calculated as the Euclidean norm $||q_{i}||={\langle q_{i}q_{i}\rangle }^{1/2}$ of the inferred parameters for a particular coupling, and therefore carries the same unit of measure as the natural frequency (Hz). The correlation $\rho_{i}\left(q_{i},q_{j}\right)=\langle {\tilde{q}}_{i}{\tilde{q}}_{j}\rangle /\left(\left||{\tilde{q}}_{i}||\;||{\tilde{q}}_{j}|\right|\right),$ of two coupling functions where ${\tilde{q}}_{i}$ are the deviations from the mean, ${\tilde{q}}_{i}=q_{i}-\langle q_{i}\rangle$, gives the similarity of their forms, irrespectively of their amplitudes (Kralemann et al., 2013). Here, we propose a further extension of this index. By calculating the correlation of a coupling function *q* with a sequence of numerically-generated forms *Q* having specific shape features, taken from a bank, one can determine which of those features is dominant in *q*. The numerical set simulates the shape of a direct coupling from the slower oscillation to the faster, phase-shifted by an angle ϑ along the 2π axes. Thus, the numerical form *Q*_ϑ_ generating the highest ρ carries dual information: the extent of the similarity (described by ρ itself) and the corresponding phase, given by ϑ. See Figure 1 and the animation video 1 in the Supplementary Material. A natural way of presenting this information is by plotting it on the complex plane to provide a polar representation of the similarity index $P_{q}=\rho_{q}e^{i\vartheta }$.

In neuroscience, the cross-frequency analyses reported to date have mostly focused on the *net* coupling. In contrast, coupling functions enable one to study the functional dependences of the distinct contributions from the individual oscillations. This procedure, referred to as *coupling decomposition*, separates a *net* pairwise coupling *q*_*i*_(ϕ_*i*_, ϕ_*j*_) into its *partial* self-coupling ${\overset{®}{q}}_{i}\left(\phi_{i}\right)$, direct-coupling ${\overset{®}{q}}_{i}\left(\phi_{j}\right)$, and common (or indirect) ${\overset{®}{q}}_{i}\left(\phi_{i},\phi_{j}\right)$ coupling components (Iatsenko et al., 2013; Stankovski et al., 2016). The inference of both net and partial coupling has been validated numerically (Stankovski et al., 2015). The direct-coupling ${\overset{®}{q}}_{i}\left(\phi_{j}\right)$ describes the influence of the direct unidirectional driving exerted by one oscillator on the other. Arguably, this is the most observed interaction in physiology, often linked to modulation mechanisms; it dominates in a number of the coupling functions discussed below. Similarly, for a triplet coupling function *q*_*i*_(ϕ_*i*_, ϕ_*j*_, ϕ_*k*_) one can decompose the self, direct, and common components depending on either one or two phase variables. Additionally, one can have the direct component ${\overset{®}{q}}_{i}\left(\phi_{j},\phi_{k}\right)$ from two phase variables exerting a joint influence, and the common component between all three phases ${\overset{®}{q}}_{i}\left(\phi_{i},\phi_{j},\phi_{k}\right)$. Generalization to higher κ-dimensional couplings is implicit. These couplings in a κ-dimensional network could reflect a joint functional influence from a cluster subnetwork.

### 2.5. EEG recordings and signal processing

The multichannel EEG recordings analyzed in this work were downloaded from the Neurophysiological Biomarker Toolbox (NBT) dataset (O'Gorman et al., 2013; Poil et al., 2013). The signals were recorded for a group of 16 subjects (of which 10 were female, median age 27 years, range 21–48) in the resting state for 8 min, with a sampling frequency of 200 Hz. During the first 4 min, subjects were asked to keep their eyes open, and in the following 4 min to keep them closed. Signals from 19 EEG electrodes corresponding to the international 10–20 system were selected from the dataset for the analysis.

The cross-frequency intervals were extracted by a standard (FIR and no-phase-shift) filtering procedure. The boundaries for the conventional frequency intervals were: delta δ = 0.8–4 Hz, theta θ = 4–7.5 Hz, alpha α = 7.5–14 Hz, beta β = 14–22 Hz, and gamma γ = 22–40 Hz. Special care was taken to minimize cardiac components and powerline interference (Lehnertz et al., 2014; Iatsenko et al., 2015). The phases of the filtered δ and α were estimated by use of the Hilbert transform, followed by the protophase-phase transformation (Kralemann et al., 2008).

### 2.6. Eyes-open and eyes-closed states

The extensive changes that the simple closing of the eyes triggers in the brain caught the attention of the very first electroencephalographers (Berger, 1933). It is now known that exclusion of visual input from the central system causes the power of brain activity to increase instantaneously across all the conventional frequency ranges (Barry et al., 2007). The most striking change occurs within the α rhythm, and it has its strongest effect on the occipital part of the scalp, over the visual cortex area. It has been argued that, with eyes open, the desynchronization of α, resulting in a lower power, might occur in order to give way to a more sophisticated pattern of information processing (Klimesch, 1999).

### 2.7. The δ-to-α coupling functions

The δ-to-α interaction reflects how δ activity, associated with deep dreamless sleep (Feinberg et al., 1987), influences the α oscillations related to information processing (Pfurtscheller and Lopes da Silva, 1999). Other findings have also suggested that the δ-to-α coupling is mostly located within the frontal regions, that it is stronger during the eyes-closed resting state (Deco et al., 2010; Jirsa and Müller, 2013), and that a strong δ-to-α link exists during non-REM sleep (Bashan et al., 2012).

Cross-frequency interactions are usually mediated by the slower oscillations modulating the faster ones (Brunel and Wang, 2003; Lakatos et al., 2005; Händel and Haarmeier, 2009). In particular, task-based studies suggest that slow oscillations, which are extended across the scalp, modulate the spatial extent of the faster oscillations, which are more localized (Palva et al., 2005; Isler et al., 2008; Canolty and Knight, 2010).

In the light of this, and because of the crucial role that the α oscillation (Klimesch et al., 2007; Eidelman-Rothman et al., 2016) plays in the eyes open (EO) and eyes closed (EC) states, we focused on the analysis of δ-to-α coupling functions. In doing so, we are able to assess, quantify, and describe in detail the functional mechanisms that define the interaction in question.

Moreover, the multichannel recordings allowed us to investigate couplings between δ and α oscillations extracted from different probes, and hence to create connectivity maps illustrating how the δ-to-α modulation differs in the EO and EC states. The coupling strength was first quantified. Note that, in earlier work (Musizza et al., 2007; Jirsa and Müller, 2013; Lehnertz et al., 2014) the use of the terms “coupling causality” and “directionality” refers to the *net* coupling strength.

### 2.8. Surrogate testing

When applying non-linear analysis techniques, one should bear in mind that the linear properties of the signals, like autocorrelation or spectral features, are likely to affect the measure. To discriminate the genuine results from the ones happened by chance, one can apply surrogate testing (Theiler et al., 1992; Schreiber and Schmitz, 1996, 2000; Paluš and Hoyer, 1998; Kreuz et al., 2004). The idea behind this technique is to apply the non-linear method in question to independent time series that have the same, or as close as possible, statistical properties as the original time signals, while randomizing the expressions of the non-linear property being measured. This procedure allows one to define a threshold beneath which any result is considered spurious.

In practice, when inferring couplings even from very weakly-coupled (or completely uncoupled) systems, the methods always detect some non-zero values of apparent coupling strength. Surrogate testing can then be used to establish the “zero-level” of apparent coupling corresponding to uncoupled signals. In order not to bias the threshold with effects due to inter-subject or inter-probe variability, we applied the surrogate techniques to the same signals for which the coupling was to be measured, and we therefore define different thresholds for different subjects, pairs of probes and states.

We generated the necessary surrogates by use of the phase-shuffling (PS) method (Schreiber and Schmitz, 2000; Jirsa and Müller, 2013). This acts on the time evolution of the phase of an oscillation, wrapped between 0 and 2π, by randomizing the sequence of full phase-periods that it contains. With this technique, the linear structures of the signals are preserved but the nonlinear properties are changed. Non-stationarities appearing within each period of the oscillations are preserved. The method was applied for each subject, state, and pair of probes, thereby providing pairs of surrogate phases (δ and α). These pairs were used as input for the Bayesian inference to compute the surrogate coupling. The significance thresholds, calculated independently for each subject and combination of probes, were then set as the mean+2 standard deviations of the resultant distributions.

### 2.9. Statistical analysis

The surrogate populations were tested for normality with the Shapiro-Wilk test, with the null hypothesis that the data come from a normal distribution of unknown mean and variance. The test rejected the null hypothesis at the 5% significance level in only 3% of the surrogates, and we therefore accepted the assumption of a normal distribution. Hence, we could test the coupling from the original signal by comparison with the significance threshold.

The non-parametric Wilcoxon paired test was used to determine the significance of differences between the EO and EC distributions for each frequency within the power spectra, for the averaged power within each frequency interval, for the coupling strength and for the similarity of coupling functions.

## 3. Results

### 3.1. Spectral analysis

**Figure 2:**
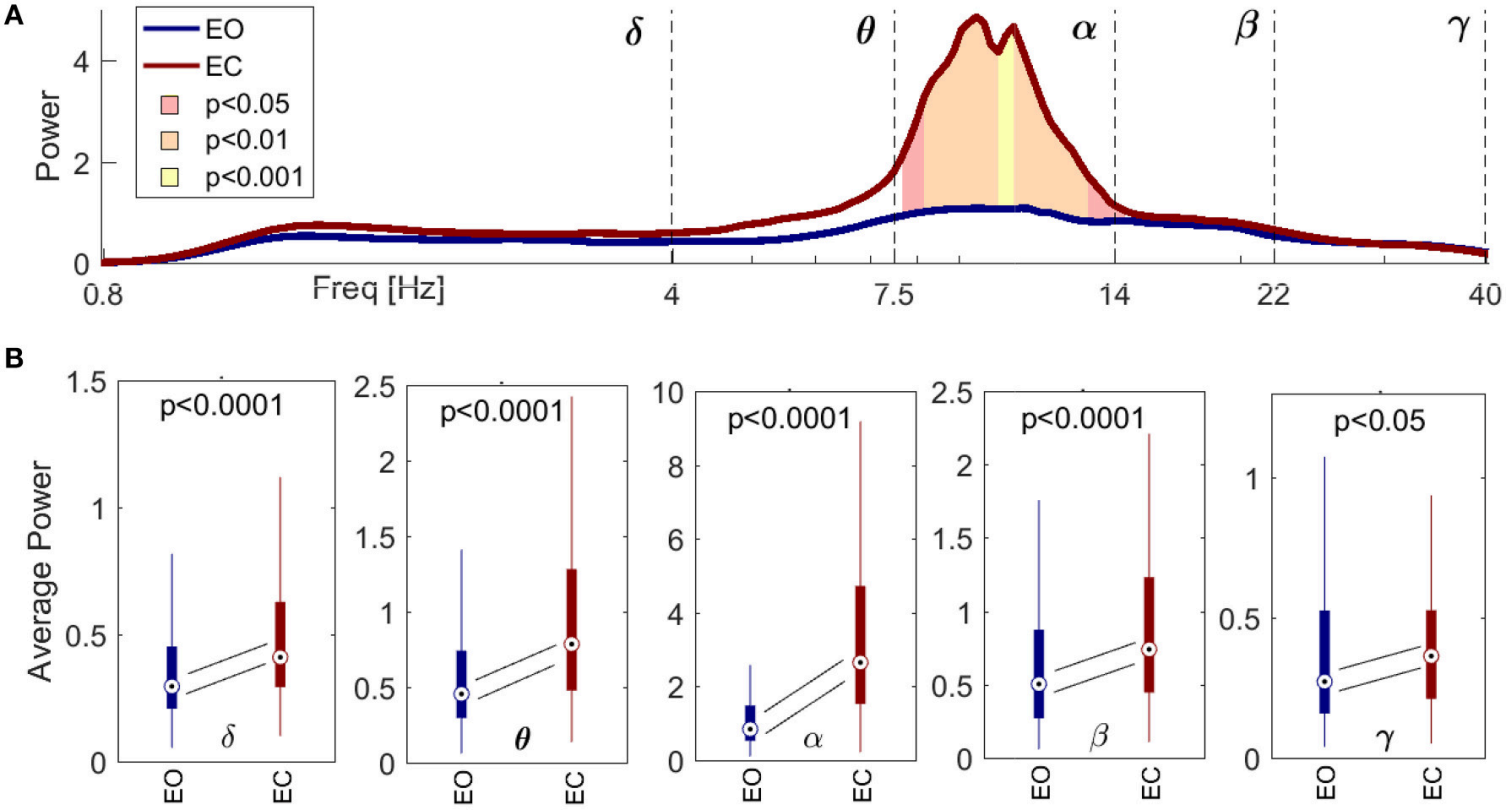
Spectral comparison between signals recorded during the eyes open (EO, red) and eyes closed (EC, blue) conditions, for all the probes from all the subjects. **(A)** Paired statistical comparison between the inter-probe average power spectra from each subject in EO and EC, respectively. The lines show inter-subject medians, and the ranges of significance are shaded pink for *p* < 0.05, orange for *p* < 0.01 and yellow for *p* < 0.001. **(B)** Boxplots for the average power within the five frequency intervals. Diagonal lines symbolize statistical analyses pairing corresponding values for every probe and subject, and follow the changes in the medians. The *p*-value is indicated in each case. Note that the significance of the power in **(A)** corresponds closely to the boundaries of the α interval, and that the power in **(B)** increases significantly between EO and EC for every frequency band.

Figure 2A shows the difference in spectral power between the EO (blue) and EC (red) states, for spectra averaged across all the probes. The shaded significance area coincides closely with the α band, indicating an increase of power in that interval for EC compared to EO. This increase was independent of scalp location. Figure 2B shows the statistical distributions of the averaged power within each frequency band, with a pairwise probe-by-probe statistical approach. The statistical analysis confirmed the increase of amplitude across all the frequency intervals when comparing EC with EO.

### 3.2. Coupling analysis

#### 3.2.1. Significance against surrogate data

**Figure 3:**
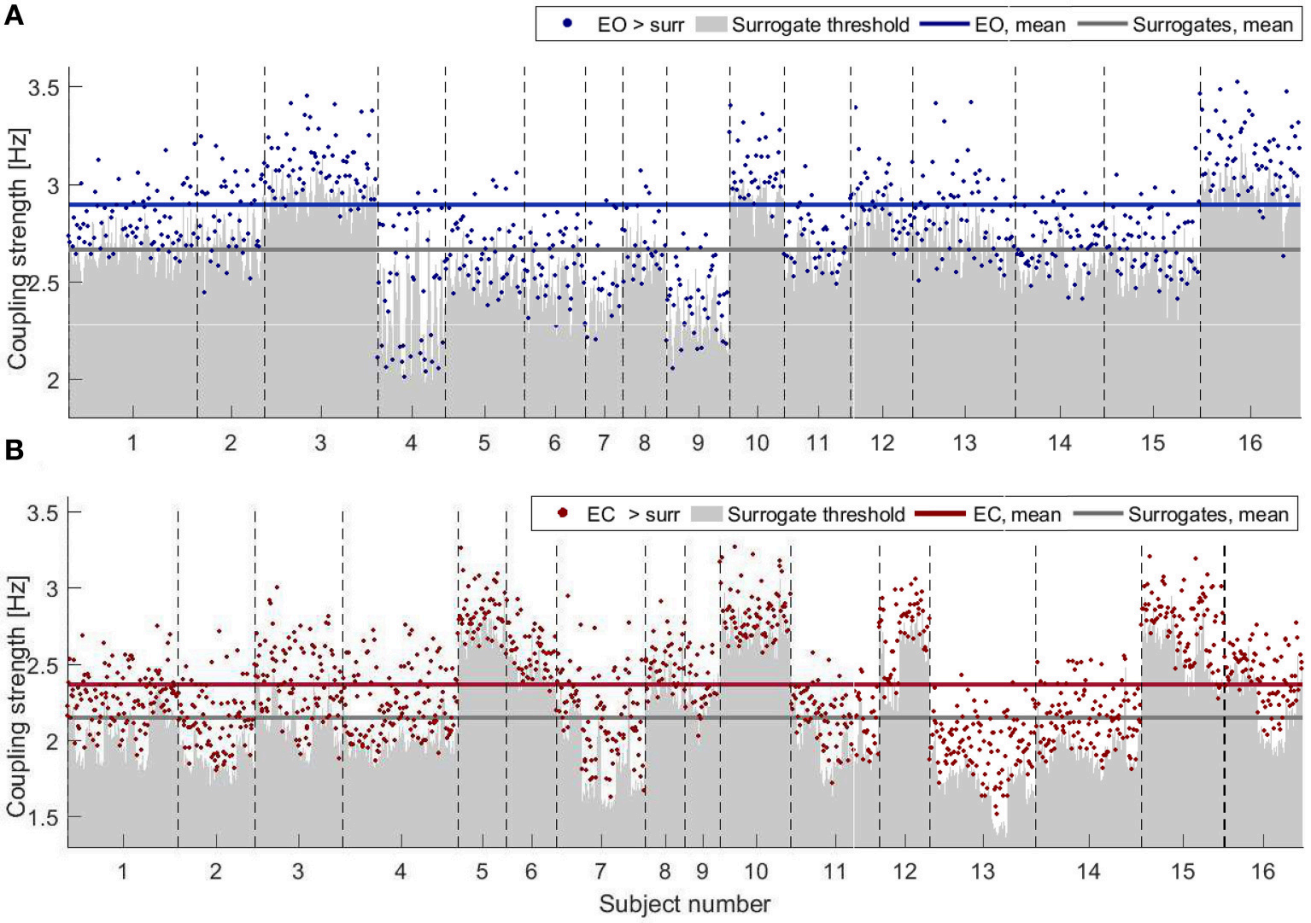
Strengths of the couplings for **(A)** EO (blue) and **(B)** EC (red) for all the subjects, shown as consecutive intervals on the *x*-axes. Only values higher than the corresponding PS surrogate threshold are shown. Couplings are selected when their strengths are higher than the mean+2STD of the corresponding surrogate distribution (gray shading). Horizontal lines indicate the average values of the surrogates and of the validated couplings (color-scheme as explained above).

Figure 3 shows the results of applying PS surrogate technique for the states of EO (in blue, Figure 3A) and EC (in red, Figure 3B). Only couplings whose δ-to-α direct-coupling strength was higher than the mean+2STD surrogate thresholds (gray shades) are indicated by dots. The average values of the surrogates and of the validated couplings (horizontal lines) are inversely proportional to the power trend, with both values for the EC being below the EO average surrogate level. For EO, however, a smaller number of probe pairs generated a coupling strength which was significant against surrogates (767 over 5,776 possible connections for EO against 1,323 over 5,776 for EC). The inter-subject variability is evident in Figure 3, where the different width of the *x*-axes portion for each subject corresponds to different number of significant connections detected.

#### 3.2.2. Inter-subject variability

**Figure 4:**
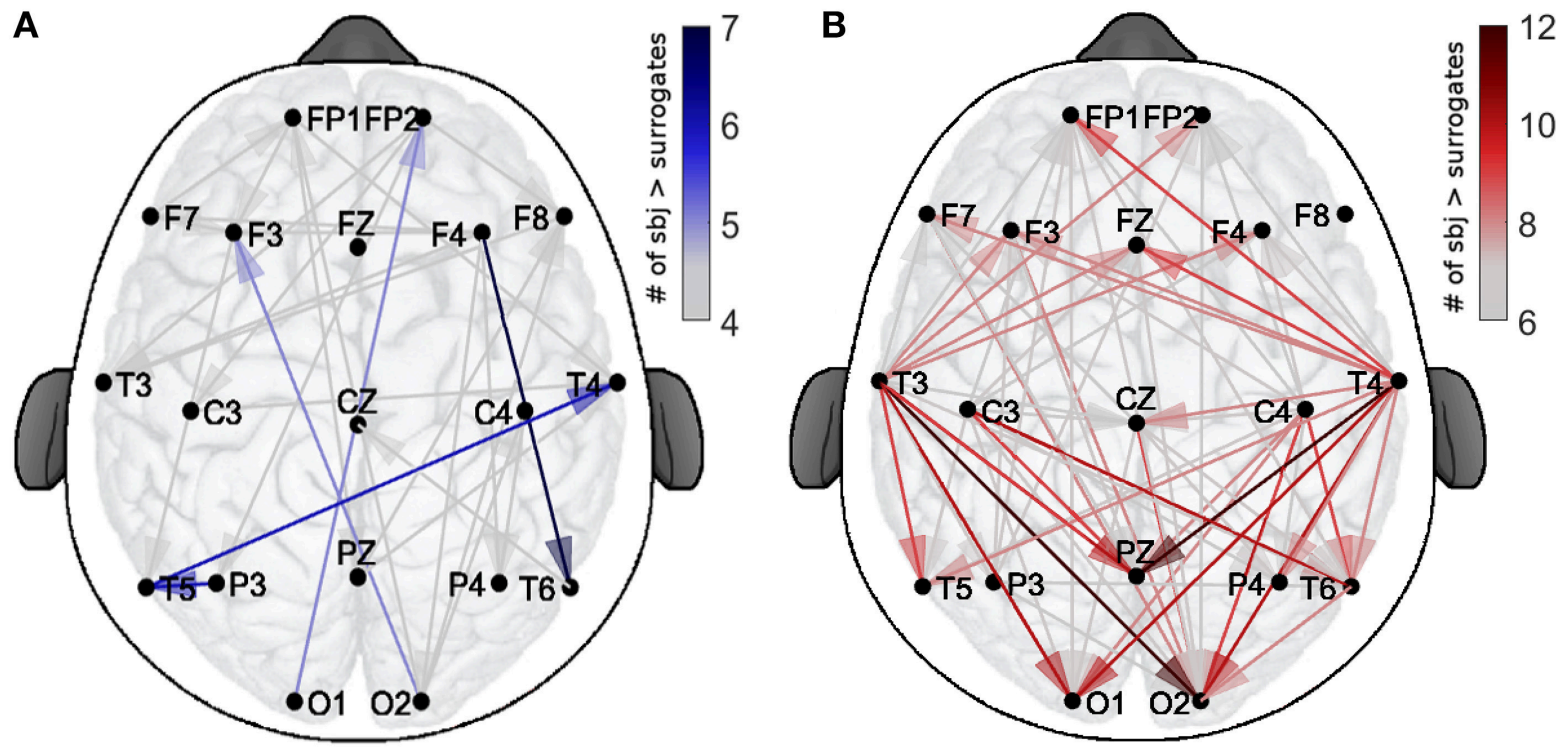
Spatial distribution of the validated coupling strengths. The color codes indicates the number of subjects with a higher direct-coupling strength than the corresponding surrogate threshold for **(A)** EO and **(B)** EC. Note the different scalings of the two color-bars, used for clarity.

In order to evaluate the spatial patterns of significant coupling, the dots shown in Figure 3 have been converted into the corresponding connections over a head-shaped map (Figure 4). The directionality of each connection is shown with an arrow starting from the probe where the δ oscillation was extracted, and ending on the corresponding location of the probe for the α oscillation.

The color-scale in Figure 4 represents the number of recurrences of significant direct coupling strength among the subjects for EO (in blue, Figure 4A) and for EC (in red, Figure 4B). For clarity of visualization, arrows corresponding to less than 4 subjects for EO (for which 767 couplings were detected as non-surrogates) and 6 for EC (for which 1,323 couplings were detected as non-surrogates) are not shown. The more intense colors correspond to larger numbers of subjects exhibiting significant coupling strength for a specific arrow for each state, e.g., 7 for EO and 12 for EC.

The figure shows how, for EO, two inter-hemispheric occipital-to-frontal δ-to-α couplings were exhibited by 5 subjects and one inter-hemispheric temporal long range connection, plus two intra-hemispheric, were detected in groups of 6 or 7 subjects. For EC, besides being in higher number, the significant connections were detected especially from temporal to occipital locations, and from temporal to the parietal Pz (for groups of 10–12 subjects). A clear pattern of temporal-to-frontal coupling was also detected, for smaller groups (8–9 subjects).

### 3.3. Coupling functions analysis

#### 3.3.1. Form of the coupling function

**Figure 5:**
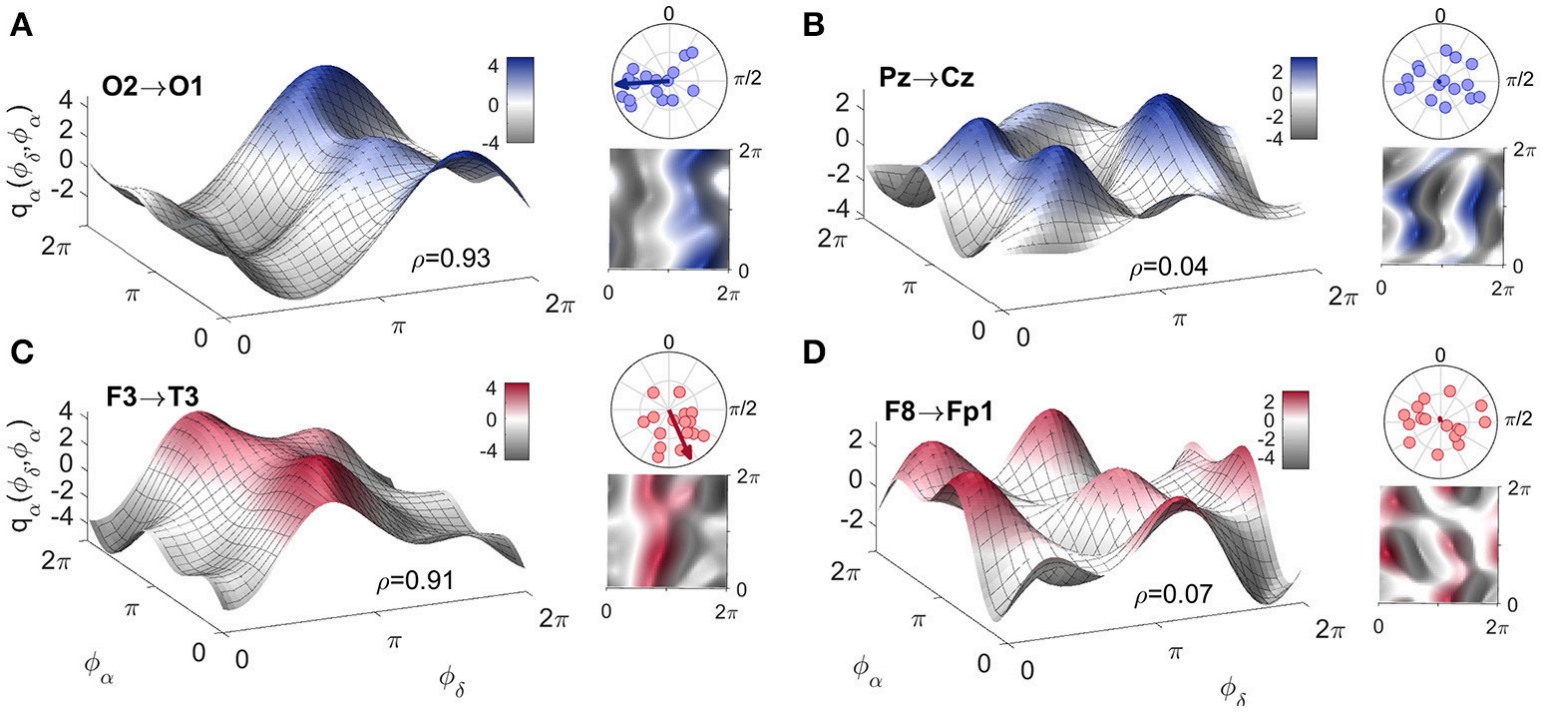
Examples of inter-subject averages of coupling functions between particular pairs of probes. They have been selected for generating **(A,C)** the highest and **(B,D)** the lowest similarity indices, as shown. The arrows in the polar plots in the top right corners of each panel indicate the similarity indices for the averaged coupling functions, while the dots indicate the similarity indices for individuals. Note that in B and D the arrows are of negligible dimension. A complementary 2D color-contour plot of the coupling function is given in the bottom right-hand corner of each panel.

To complement the coupling strength analysis, we now focus on the coupling functions themselves and discuss their unique properties. The results are summarized in Figure 5. The panels show the coupling functions corresponding to the links having the highest and lowest similarity indices for the intersubject average, for EO and EC. First, we describe in detail the δ-to-α coupling function as a 3D surface characterizing the EO state, as shown in Figure 5A. The form of this function indicates that much of the δ-to-α coupling is attributable to the direct contribution of the δ oscillation. It has a sine-like waveform along the ϕ_δ_-axis, but is mostly constant along the ϕ_α_-axis. This reveals the underlying functional mechanism i.e., shows that, when δ oscillations are between π and 2π, the sine-wave coupling function is higher and the δ activity accelerates the α oscillations; similarly, when the δ oscillations are between 0 and π, the coupling function is decreased and δ decelerates the α oscillations. The highest acceleration i.e., the ridge of the 3D function plot is around 3π/2. The form of the coupling function of Figure 5C for the EC state is similar to the one for EO, but it is shifted with the highest acceleration being between 0 and π. In contrast to these two, the coupling functions shown in Figure 5B for EO and Figure 5D for EC, have uncharacteristic and undefined rippled form of lower amplitude.

These qualitative observations can be quantified and presented in terms of the polar similarity index. In Figure 5 these are shown as a circle-map in the top-right corner of each plot. For the polar similarity index of EO (Figure 5A) one can note that the values for individual subjects (the dots in the circle-map), are distributed around a certain direction, and that the arrow for the average similarity index has the quite high value of 0.93. Also, the direction of the average arrow has an angle of about 3π/2, which is the ridge of the average coupling function for the highest acceleration of α oscillations (compare the 3D plot in Figure 5A). The polar similarity index for the EC state (Figure 5C) shows a similar trend, with a high index of 0.91, but a different arrow direction. For the least-similar forms (Figures 5B,D) the similarity indices are very low with moduli close to zero (the dots are distributed sparsely), leading to almost unnoticeably small arrows at the center of the circle. Because these coupling functions come from inter-subject averages, it can be seen how the plot of polar similarity indices explains not only their morphology, but also their origin and the inter-subject variability.

#### 3.3.2. Time-variability of neural coupling functions

**Figure 6:**
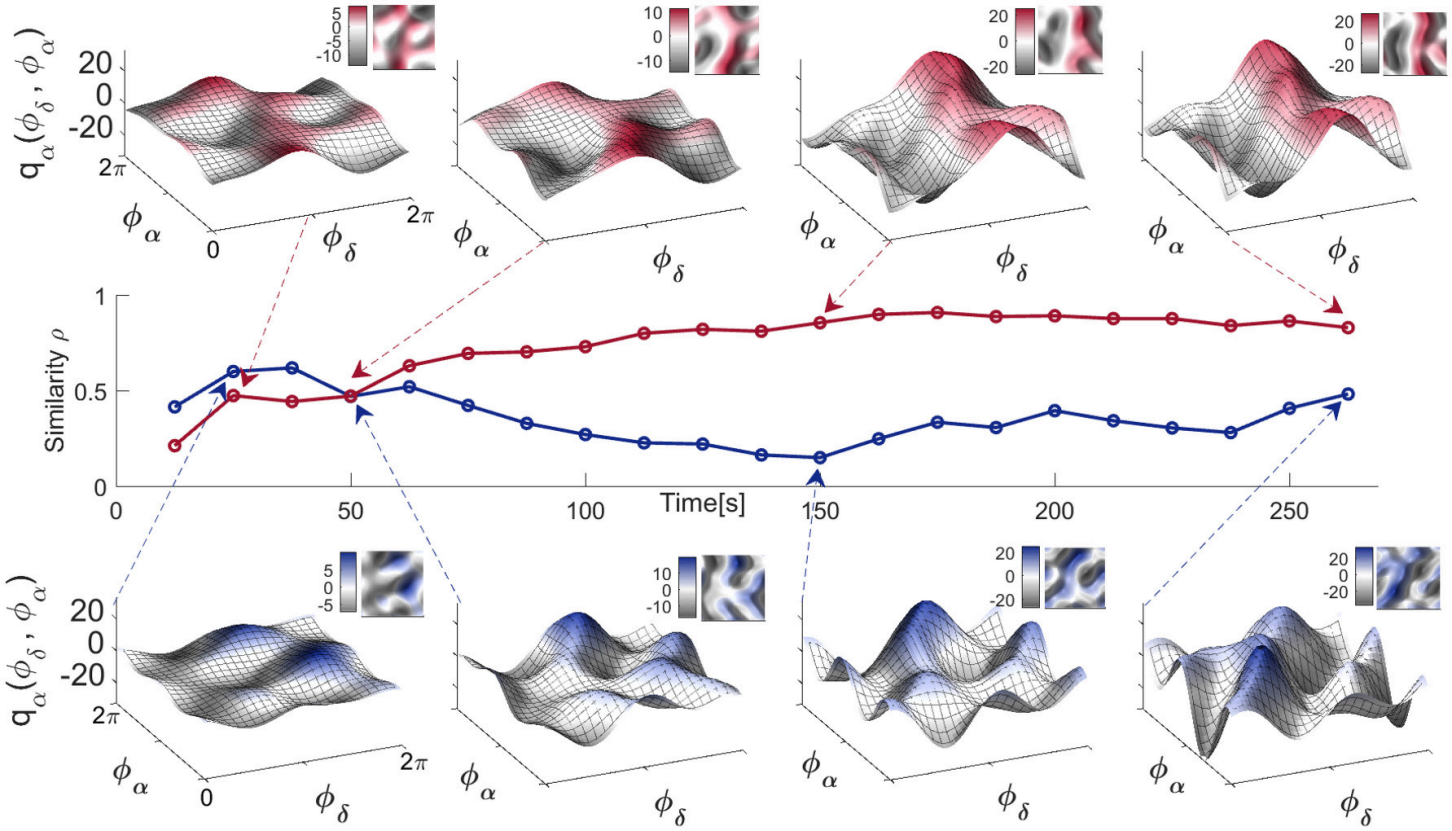
Time-evolution of the δ-to-α coupling functions in the resting state. Middle panel: Time-evolution of the similarity index ρ_α_(δ, α) for the EO and EC states of a single representative subject. Top panel: The δ-to-α coupling functions for EC inferred at four particular moments in time, as indicated by the arrows. Bottom panel: The δ-to-α coupling functions for EO inferred at four particular moments in time. Complementary 2D color-contour plots of the coupling functions are given in the top right-hand corner of their respective panels.

Physiological systems and processes, including neural oscillations, do not exist in isolation. They can be affected by a variety of external influences making their dynamics, to a greater or lesser extent, time-varying. In such cases, one can use the dynamical Bayesian method to infer time-varying neural dynamics, as demonstrated in Figure 6. The coupling functions for the EC state in Figure 6 (top), inferred at four different times, show that not only the strength but also the form of the coupling functions can vary in time. This time-variability is a representative example and it was not correlated with the coupling function time-variability of other subjects' EEG signals. It is more pronounced for the four EO coupling functions in Figure 6 (bottom), which vary even more. Consequently, the similarity index Figure 6 (middle) which quantifies the effect is also time-varying, with higher values for the EC state resulting in more-similar forms of coupling function—compare for example the last two coupling functions in Figure 6 (top). This time-variability and the evolution of the resting state δ-to-α coupling functions can be appreciated even better through the animation video 2 in the Supplementary Material, generated for each of the times in Figure 6.

### 3.4. Quantitative group analysis

To investigate the quantitative statistics of each group of subjects we calculated the average values of the significant coupling strengths, with the corresponding surrogates' value subtracted, and the moduli of the polar similarity indices for the coupling functions of all the links for each subject. Then we compared statistically the distributions of these values for the two groups of subjects. To present the differences between the distributions visually, we use standard boxplots which refer to the descriptive statistics (median, quartiles, maximum and minimum).

**Figure 7:**
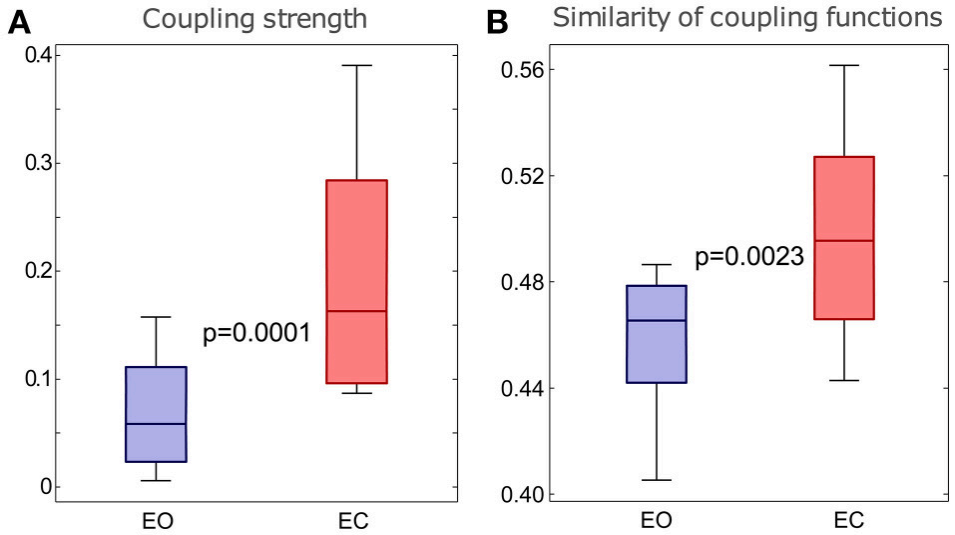
Differences in the δ-to-α coupling strength above surrogates **(A)** and in the similarity of form of the δ-to-α coupling functions **(B)** for the two groups of subjects with EO and EC. The *p*-values indicated within each panel represent the statistical differences between the EO and EC states. Whiskers indicate ±2.7 standard deviations of the distribution.

The results in Figure 7 show that there were statistically significant differences for both the coupling strengths and the similarity of coupling functions between the EO and EC states. Figure 7A shows that the coupling strengths detected for the EC is significantly higher than the EO. Similarly, Figure 7B shows that the similarity index for δ-to-α coupling functions for the EC were significantly higher than for the EO. The latter also means that there was larger variability of the coupling functions for the EO state, compared to the EC state. Overall, the similarity of coupling functions for the EO and EC states was not very high (in the interval of [0,1]), indicating that there is relatively high variability of coupling functions for both of the resting states.

## 4. Discussion

### 4.1. The EO and EC resting states

Much has already been done, mostly through fMRI and EEG analysis, to demonstrate the existence of resting state interactions, including the formation and dissolution of resting state functional network configurations around a stable anatomical connectivity (Berger, 1933; Barry et al., 2007; Deco et al., 2010; Jirsa and Müller, 2013; O'Gorman et al., 2013). Our application of coupling functions to the resting state revealed the underlying mechanisms of interaction and has identified a number of differences between the EO and the EC states.

As there were more significant couplings in the EC than in the EO state (Figure 3), it is obvious that there will be more coupling links for the EC than for the EO state when presented spatially (Figure 4). What is interesting is that, for EC, different subjects seem to have a preferential pattern of directions, with the δ oscillation from the anterior temporal lobes (probes *T*3 and *T*4) acting as “hubs,” influencing the phase of α in both the frontal and occipital directions. The occipital probes *O*1 and *O*2 are the most susceptible to the difference in α power (Figure 2) as they are placed over the visual area of the cortex. In EO, they act as a starting point for δ modulating long range connections toward the frontal cortex, which existed in five subjects, and then disappeared in EC. In contrast, for EC these probes receive the influence in their α rhythm from temporal and central probes.

The δ-to-α coupling functions had a specific shape, showing that the coupling is predominantly like a direct sine wave due to the δ influence, which accelerates and decelerates the α oscillations. Importantly, the form was similar for the EO and EC states (Figure 5), with distinctive variations and shifts along the δ oscillation. This similarity implies that the same underlying interaction mechanism exists in the EO and EC states, and that the difference between these two resting states corresponds to increasing and decreasing some of the connection strengths (or to switching them on-off).

Because we reconstructed the form of the coupling functions, we were able to observe what they look like for both individual and averaged connections and subjects. Even though we found relatively similar forms of function, we also observed a certain degree of variability, both inter-subject variability (Figure 5) and time variability (Figure 6) of the form. These should be taken into account when average values are used, for example in making multi-subject statistics.

Finally, for the comparison of the EO and EC states (Barry et al., 2007) our analysis confirmed that the spectral power of the α oscillations in EC is significantly larger than that of EO (Klimesch, 1999). It also showed that there are a larger number of real (i.e., validated by surrogate testing) δ-to-α couplings for the EC state (Jirsa and Müller, 2013), that the form of the coupling functions was similar for EO and EC, that the coupling functions were somewhat less variable for EC than for EO, and that this dominance of the EC state in the interactions was confirmed also by the quantitative boxplot statistics for the whole groups of subjects.

### 4.2. Methodological aspects and generalizations

The assessment of neural coupling functions through the phase dynamics of interacting neural oscillations enables us to study their acceleration/deceleration, i.e., timing and coordination. The generalization to amplitude coupling functions is implicit. In such cases, one should be able to determine a plausible state model in relation to the dimensionality of the signals. Amplitude neural coupling functions can reveal the mechanism through which the strength and power of one neural oscillation are affected by the influence of the other oscillations.

Earlier effective connectivity methods for the inference of neural interactions have in principle contained coupling functions within their models of the interacting dynamical systems. The question we address here, in addition to presenting an efficient Bayesian method for determination of coupling functions, is that of how to *assess* the neural coupling functions. We have shown how to unify a functional unit which can be quantified and compared with other such units, and whose evolution can be followed in time. The key characteristic that distinguishes this assessment is the form of the neural coupling functions. A unified and effective coupling function analysis can provide insights that go far beyond just knowing that neural interactions exist.

The pairwise investigation can further be generalized to higher degrees of network complexity (Kralemann et al., 2014; Stankovski et al., 2015). One might, for example, study the coupling functions between the brain and other physiological oscillations, forming a physiological network (Musizza et al., 2007; Stefanovska, 2007; Bashan et al., 2012; Stankovski et al., 2016). The brain is a heavily connected network (Park and Friston, 2013) and coupling functions could be applied to reveal the functional mechanisms operative at different levels and sublevels of the interactions. In network topology with nodes and edges (Albert and Barabási, 2002) this would mean that, not only could the existence, strength and direction of the edge be studied, but also the underlying functional mechanism giving rise to the edge. The multivariate coupling function assessment can then be linked to hypergraphs (Karypis and Kumar, 2000; Zass and Shashua, 2008), though it was argued recently that, for larger networks (*N* > 10), there is no significant benefit from using multivariate inference of coupling (functions) and partialization (Rings and Lehnertz, 2016).

The time-varying form of the coupling functions (Figure 6) can be a cause of self-organization transitions, like the emergence of network clustering, or of the systems going into-and-out-of synchronization (Stefanovska et al., 2000; Varela et al., 2001), even for an invariant net coupling strength (Stankovski, 2017). More importantly, having detected and characterized a neural coupling function, one can then use this knowledge to detect, or even to predict, the onset of phase synchronization (Kiss et al., 2005). In such cases, the key feature is the known form of the coupling function which, depending on parameters like frequency, coupling strength, or polar similarity index, can predict the synchronization transition. This could have important implications for the prediction of epileptic seizures (Lehnertz and Elger, 1998; Fell et al., 2001) which occur or disappear as synchronous activity in the brain.

#### 4.2.1. Limitations

The limitations of the method should also be borne in mind. First, the whole analysis starts with the extraction of one-dimensional vectors of phases from data which probably have a non-trivial distribution of spectral content. Especially when the coupling mode is extracted from a single signal, the filtering must be done with extreme care: spillage between different frequency intervals, as well as splitting of one mode into two intervals, will result in an artificial “common” coupling. Whenever bandpass-filtering is involved, one should exclude the possibility of investigating high-to-low frequency coupling, because any modulation of the lower frequency due to the phase of the higher one will probably be erased from the filtered mode. In any case, these couplings will usually turn out to be insignificant compared to surrogates later in the analysis.

The windowed nature of dynamical Bayesian inference carries its own limitations, too, as the length of the window is fixed for every computation. This parameter must be chosen with care, and should be adjusted so as to include a sufficient number of periods of the lower frequency involved. We found that 6–10 periods is a reasonable lower limit for this number. Due to the uninformative flat prior used for the initial window, the resultant inference of the first window should be interpreted with care. Moreover, the signals' own particular features must also be taken into account: a high degree of time-variability would need a correspondingly shorter window for the dynamical inference to follow the evolution correctly. If the method is to be generalized for use other than with a phase dynamics model, one should be careful not to infer dynamics due to non-specific, non-stationary, processes instead of genuine coupling.

### 4.3. Conclusion

In conclusion, coupling functions bring a novel perspective to neuroscience that is unique in that it provides access to the functional *form* of a coupling. The polar similarity index that we have introduced allows one to describe the form in quantitative detail. The comparisons of δ-to-α phase-to-phase coupling functions in the EO and EC resting states demonstrate how neural coupling functions can be reconstructed from spatially distributed sources, and what benefits and possibilities are introduced by their assessment. We have confirmed the previous result that the direct coupling is stronger during EC, and we have shown for the first time that the coupling function is significantly less variable in that state. The EO/EC states were taken as an example on which to base a discussion of methodological issues and, in so doing, to point to the wider implications and possibilities of the method itself. One may hope to gain new insights into the neuronal mechanisms underlying certain diseases from studies of coupling functions. In principle, the method can equally be applied to the time series created by *any* pair of coupled oscillatory processes.

## Author contributions

TS and VT did the analysis. TS and VT wrote the draft of the paper assisted by PM. AS planned and oversaw the entire enterprize. All authors edited the text and contributed ideas and content.

### Conflict of interest statement

The authors declare that the research was conducted in the absence of any commercial or financial relationships that could be construed as a potential conflict of interest.
