# Supplementary material for: Neural Cross-Frequency Coupling Functions
Source: Front Syst Neurosci. 2017 Jun 15;11:33. doi: 10.3389/fnsys.2017.00033 (PMC5471314; doi:10.3389/fnsys.2017.00033)
Supplement: Supplementary file 3 [file DataSheet1.pdf]

APPENDIX

Dynamical Bayesian inference – technical details

The network model of N coupled phase oscillators Eq. (2) is to be inferred by a dynamical Bayesian approach (Smelyanskiy et al., 2005; Stankovski et al., 2012). The model in Eq. (2) can be approximated with a series of Fourier components $\mathbf{\Phi}$ as

$$\dot{\phi}_i(t) = \mathbf{c}_0^{(i)} + \sum_{k=-K}^{K} \mathbf{c}'^{(i:l)}_k \mathbf{\Phi}_k(\phi_l) + \sum_{k=-K}^{K} \mathbf{c}''^{(i:l,m)}_k \mathbf{\Phi}_k(\phi_l, \phi_m) + \\ + \sum_{k=-K}^{K} \mathbf{c}'''^{(i:l,m,n)}_k \mathbf{\Phi}_k(\phi_l, \phi_m, \phi_n) + \ldots + \xi_i(t).$$

The aim of the inference is to compute the set of parameters $\mathbf{c}$ and the noise diffusion matrix $\mathbf{D}$ characterizing the network, starting from the phase dynamics extracted from the time-series $\mathbf{x}$.

Set $\mathcal{M} = \{c_k^{(i)}, D_{i,j,\ldots}\}$ and $\mathcal{X} = \{\mathbf{x}_l \equiv \mathbf{x}(t_l)\}$ ($t_l = lh$), with $l = 1, \ldots, L$ being L the number of samples of the time series, Bayes' theorem allows one to obtain the *posterior* density $p_{\mathcal{X}}(\mathcal{M}|\mathcal{X})$ of the unknown matrix of parameters $\mathcal{M}$ from $\mathcal{X}$, given a *prior* density $p_{\text{prior}}(\mathcal{M})$ (based on observations and representing previous knowledge of the unknown parameters), by building a *likelihood* function $\ell(\mathcal{X}|\mathcal{M})$:

$$p_{\mathcal{X}}(\mathcal{M}|\mathcal{X}) = \frac{\ell(\mathcal{X}|\mathcal{M})\, p_{\text{prior}}(\mathcal{M})}{\int \ell(\mathcal{X}|\mathcal{M})\, p_{\text{prior}}(\mathcal{M}) d\mathcal{M}}.$$

Because the noise is treated as white and Gaussian, i.e. statistically independent, the likelihood at each time is considered as a product over l of the probability of observing $\phi_{i,l+1}$. The likelihood function is computed through the stochastic integral of the noise term over time. The joint probability density of the phase dynamics process in respect of $[\phi_i(t_{l+1}) - \phi_i(t_l)]$ is calculated using the joint probability density of z_i by imposing $P[\phi_i(t_{l+1})] = \det(J_\xi^\phi)P(\xi)$, where J_ξ^ϕ is the Jacobian term of the transformation of variables that can be calculated from the base functions $\Phi_{i,k}$.

From this, the minus log-likelihood function $S = -\ln \ell(\mathcal{X}|\mathcal{M})$ can be expressed as

$$S = \frac{L}{2} \ln |\mathbf{D}| + \frac{h}{2} \sum_{l=0}^{L-1} \Big(\mathbf{c}_k \frac{\partial \mathbf{\Phi}_k(\phi_{\cdot,l})}{\partial \phi} + \\ + [\dot{\phi}_l - \mathbf{c}_k \mathbf{\Phi}_k(\phi^*_{\cdot,l})]^T (\mathbf{D}^{-1}) [\dot{\phi}_l - \mathbf{c}_k \mathbf{\Phi}_k(\phi^*_{\cdot,l})] \Big),$$

where summation over the repeated indices k is implicit, and the dot index in $\phi.$ is substituted with the relevant index.

Assuming that the prior probability of parameters $\mathcal{M}$ is a multivariate normal distribution, and taking into account the quadratic form of the log-likelihood (Eq.4), the posterior probability will also be a multivariate normal distribution. With this particular distribution for the parameters $\mathbf{c}$, with mean $\bar{\mathbf{c}}$, and covariance matrix $\mathbf{\Sigma}_{\text{prior}} \equiv \Xi^{-1}{}_{\text{prior}}$, the stationary point of S is calculated recursively (Smelyanskiy et al., 2005) using the equation

$$\begin{aligned}
\mathbf{D} &= \frac{h}{L}\left(\dot{\phi}_l - \mathbf{c}_k \mathbf{\Phi}_k(\phi^*_{\cdot,l})\right)^T \left(\dot{\phi}_l - \mathbf{c}_k \mathbf{\Phi}_k(\phi^*_{\cdot,l})\right), \\
\mathbf{r}_w &= (\Xi_{\text{prior}})_{kw}\,\mathbf{c}_w + h\,\mathbf{\Phi}_k(\phi^*_{\cdot,l})\,(\mathbf{D}^{-1})\,\dot{\phi}_l + \\
&\quad - \frac{h}{2}\frac{\partial \mathbf{\Phi}_k(\phi_{\cdot,l})}{\partial \phi}, \\
\Xi_{kw} &= (\Xi_{\text{prior}})_{kw} + h\,\mathbf{\Phi}_k(\phi^*_{\cdot,l})\,(\mathbf{D}^{-1})\,\mathbf{\Phi}_w(\phi^*_{\cdot,l}), \\
\mathbf{c}_k &= (\Xi^{-1})_{kw}\,\mathbf{r}_w,
\end{aligned}$$

where the summations over $l = 1, \ldots, L$, and over the repeated indices k and w, are implicit.

The posterior multivariate probability $\mathcal{N}_{\mathcal{X}}(\mathbf{c}|\bar{\mathbf{c}}, \Xi)$ is computed by applying Eqs.5: it explicitly defines the probability density of each parameter set of the model (Eq. 3).

This inference technique is applied to the information provided by a stream of sequential blocks coming from the time-series. The current distribution (Eq.5) is computed, based on the evaluation of the previous block of data, i.e. informative *priors* are used. At each iteration the current *prior* depends on the previous *posterior*; because the first initial *prior* does not contain any information, it is set to a flat normal distribution with $\Xi_{\text{prior}} = 0$ and $\bar{\mathbf{c}}_{\text{prior}} = 0$.

To handle dynamics characterized by time-variable interacting dynamics, explicit information propagation is used between consecutive blocks of data (Stankovski et al., 2012): the covariance matrix of the next prior is computed by convolution of the current posterior with the current diffusion matrix Σ^l_{diff}. This measure describes how much the parameters can change: $\Sigma^{n+1}_{\text{prior}} = \Sigma^n_{\text{post}} + \Sigma^n_{\text{diff}}$.
